# Distribution of *Rickettsia* spp. and *Bartonella* spp. in questing *Ixodes ricinus* ticks in Norway

**DOI:** 10.1016/j.ijppaw.2026.101271

**Published:** 2026-08-03

**Authors:** Hanne Kloster, Camilla Stormo, Andrew Jenkins, Arnulf Soleng, Åshild Kristine Andreassen, Vivian Kjelland

**Affiliations:** a Department of Natural Sciences, Faculty of Engineering and Science, University of Agder, Kristiansand, Norway; b Department of Natural Science and Environmental Health, University of South-Eastern Norway, Bø, Norway; c Department of Virology, Division for Infection Control and Environmental Health, Norwegian Institute of Public Health, Oslo, Norway; d Department of Pest Control, Division for Infection Control and Environmental Health, Norwegian Institute of Public Health, Oslo, Norway

**Keywords:** Tick-borne pathogens, Pathogen prevalence, Vector-borne diseases, Tick surveillance

## Abstract

Ticks are important vectors of zoonotic pathogens in Europe, but knowledge regarding the presence of *Rickettsia* spp. and *Bartonella* spp. in questing *Ixodes ricinus* in Norway remains limited. This study investigated the occurrence of these bacteria in ticks collected across the Norwegian distribution range of *I. ricinus.*

A total of 564 adult ticks and 3930 nymphs (393 pools) collected in seven counties were analysed by real-time PCR. *Rickettsia* spp. were detected only in adult ticks, with an overall prevalence of 3.9 % (22/564). Infected ticks were identified in only two counties, Østfold and Agder, with a prevalence of 11.2 and 10.9 %, respectively. Species-specific analysis confirmed *R*. *helvetica* in 77.3 % (17/22) of the positive samples. *Rickettsia* spp. were not detected in any nymph pools. *Bartonella* spp. were detected in 0.5 % (3/564) of adult ticks, representing the first report of *Bartonella* spp. in questing *I. ricinus* in Norway. Infected ticks were found in three counties, Østfold, Telemark, and Nordland. *Bartonella* spp. were not detected in any nymph pools.

These findings demonstrate that *Rickettsia* spp. and *Bartonella* spp. are present in questing adult ticks in Norway, highlighting the importance of continued surveillance of emerging tick-borne pathogens in northern Europe.

## Highlights

• Survey of *Ixodes ricinus* across its geographical range in Norway
• First detection of *Bartonella* spp. in questing ticks in Norway
• Low overall pathogen prevalence, but local *Rickettsia* spp. hotspots reached 11 %
• *Rickettsia helvetica* was the dominant *Rickettsia* species detected
• Northward tick expansion underscores surveillance needs

## Graphical abstract

**Figure:**
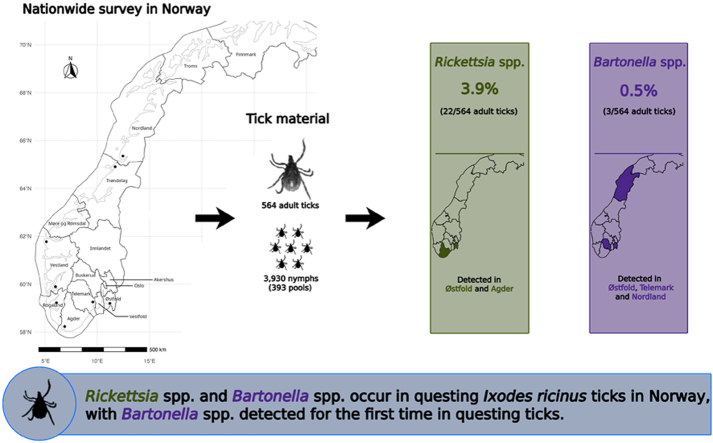

## 1 Introduction

In Norway, *Ixodes ricinus* is the most widespread tick species and plays a central role in the transmission of several emerging infectious agents (Kjelland et al., 2018; Soleng et al., 2018). Its distribution is largely confined to coastal regions, extending from Østfold County in southeastern Norway to Nordøyvågen on the island of Dønna in Nordland County (66.2204° N, 12.59° E), where the northernmost established *I. ricinus* populations have been recorded (Hvidsten et al., 2014, 2020; Mehl, 1983; Soleng et al., 2018). However, climate change is expected to lead to higher temperatures, more precipitation, and a longer growing season in Norway (Ketzler et al., 2021). These are key factors for *I. ricinus* persistence (Estrada-Pena, 2026) and are likely to contribute to increased tick abundance and geographical expansion in this region.

Ticks are important vectors of a wide range of zoonotic pathogens in Europe, including the lesser-known bacteria of the genera *Rickettsia* and *Bartonella* (Dietrich et al., 2010; Heyman et al., 2010). The order *Rickettsiales* comprises gram-negative, obligate intracellular bacteria, including the genera *Rickettsia*, *Ehrlichia, Neoehrlichia,* and *Anaplasma* (Castelli et al., 2024; Dumler et al., 2001). Within this order, members of the spotted fever group (SFG) *Rickettsia* are primarily transmitted by ticks and encompass over 30 recognized species worldwide (Dobler and Pfeffer, 2012; Guccione et al., 2023). These bacteria are maintained in enzootic cycles involving ticks and various vertebrate hosts, with transmission to humans occurring through tick bites (Parola et al., 2005). Several SFG *Rickettsia* species have been detected in Europe, including *R*. *helvetica, R*. *slovaca*, *R*. *raoultii*, and *R*. *monacensis*, with *I. ricinus* serving as an important vector and reservoir in many regions (Nelson et al., 2024; Parola et al., 2005). In Norway, *R. helvetica* is currently the only tick-borne *Rickettsia* species confirmed in questing ticks (Hvidsten et al., 2020; Kjaer et al., 2020; Quarsten et al., 2015). *R. helvetica* infections are most often asymptomatic or associated with mild, self-limiting illness presenting with non-specific symptoms such as fever, headache, myalgia, or rash in humans (Lindblom et al., 2016; Ocias et al., 2020; Oteo and Portillo, 2012). Nevertheless, the bacterium has also been detected in cerebrospinal fluid from patients with meningitis of uncertain aetiology, and has been implicated in other severe manifestations, including two cases of sudden cardiac death associated with perimyocarditis reported in Sweden (Nilsson et al., 1999, 2010). Although previous studies have confirmed the presence of *R. helvetica* in questing *I. ricinus* ticks in Norway, these investigations have been conducted over a limited geographical range (Quarsten et al., 2015; Kjaer et al., 2020), and further studies are needed to assess the geographical distribution of the pathogen. No clinical cases of rickettsiosis have been reported in Norway so far; however, a serological survey conducted in a municipality in southern Norway, where ticks are abundant, found that IgG antibodies against *R. helvetica*/*R. conorii* were present in 4.2 % of the adult population (Thortveit et al., 2020). Although these findings do not confirm the presence of *R. conorii* due to potential serological cross-reactivity among SFG *Rickettsia*, they suggest that human exposure to SFG *Rickettsia* may occur more frequently than reflected by reported clinical cases. This highlights the need for improved knowledge of the prevalence, geographic distribution, and pathogenic potential of *Rickettsia* spp. in Norway.

In contrast to *Rickettsia* spp., *Bartonella* spp. have so far primarily been identified in Norwegian wildlife and their associated ectoparasites. Here, *Bartonella* spp. have been detected mainly in wild cervids, most notably in moose (*Alces*), roe deer *(Capreolus capreolus),* and red deer (*Cervus elaphus*) (Duodu et al., 2013; Razanske et al., 2018; Sacristan et al., 2021), and in hematophagous arthropods that feed on these hosts, including deer ked (*Lipoptena cervi*) and feeding ticks removed from cervids (Sacristan et al., 2021). *Bartonella* spp. are fastidious, facultative intracellular bacteria increasingly recognized as emerging zoonotic pathogens in both humans and animals (Boulouis et al., 2005). A wide range of domestic and wild animals serve as reservoir hosts for *Bartonella* spp., while transmission most commonly occurs via arthropod vectors such as fleas, lice, sandflies, and, potentially, ticks (Billeter et al., 2008; Chomel et al., 2006). Several *Bartonella* species are associated with human disease, including *B. henselae* (cat-scratch disease), *B. quintana* (trench fever), *B. bacilliformis* (Carrison's disease), and *B. ancashensis* (associated with verruga peruana) (Billeter et al., 2008; Edvinsson et al., 2021; Karem et al., 2000). Among these, *B. henselae* is recognized as a major zoonotic pathogen globally, especially in areas with high densities of domestic cats and cat fleas (Álvarez-Fernández et al., 2018). In addition to cat-scratch disease, it has also been linked to more severe manifestations (e.g. neuroretinitis), lymphadenopathy, and infective endocarditis (Anderson and Neuman, 1997; Chomel et al., 2006). The expanding spectrum of clinical syndromes and the potential for chronic, difficult-to-diagnose infections underscore the importance of surveillance and further research into *Bartonella* epidemiology and transmission dynamics.

Despite the detection of *Rickettsia* spp. in host-seeking ticks and *Bartonella* spp. in ticks feeding on cervids in Norway, knowledge about their prevalence and distribution in questing *I. ricinus* ticks remains limited, representing a notable epidemiological knowledge gap. Hence, increased surveillance of ticks and tick-borne pathogens, particularly given the expanding distribution of *I. ricinus* in northern latitudes, is important. This study aimed to investigate the occurrence of *Rickettsia* spp. and *Bartonella* spp. in questing *I. ricinus* collected throughout the tick's geographical distribution range in Norway.

## 2 Methods

### 2.1 Study area and tick collection

**Fig. 1:**
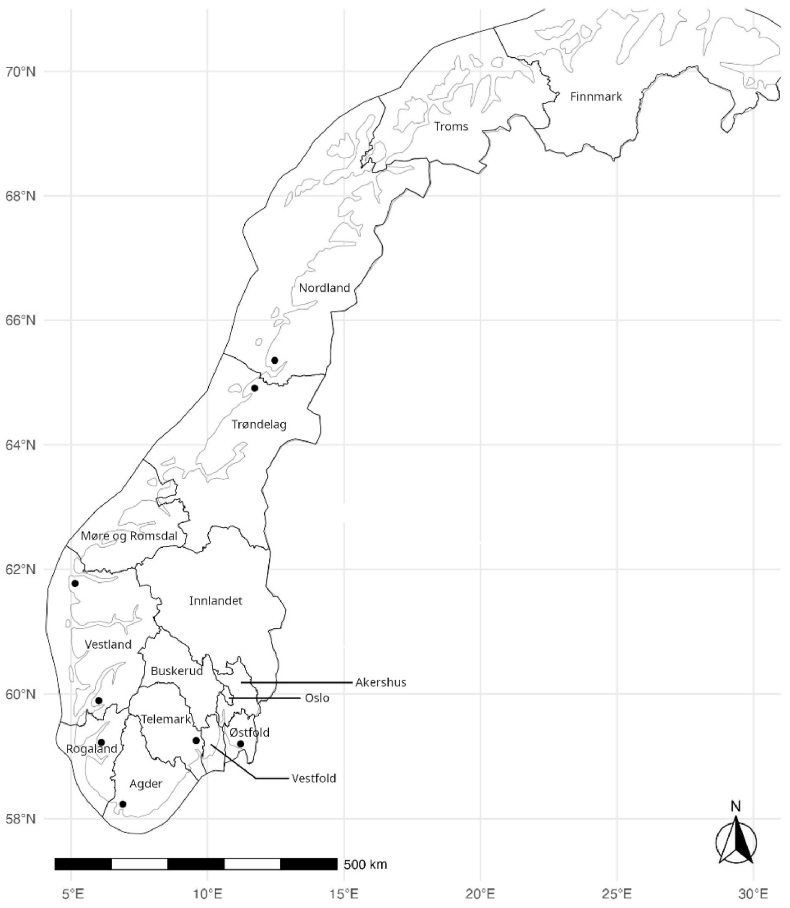
*I. ricinus* was collected along the coast of Norway, from Østfold in the southeast to Nordland in the northwest. Dots indicate sampling sites in the counties of Østfold, Telemark, Agder, Rogaland, Vestland, Trøndelag, and Nordland.

Questing *I. ricinus* were collected during 2014 – 2016 using the flagging method as previously described (Soleng and Kjelland, 2013). Ticks were collected from 7 counties along the Norwegian coast (all sites <10 km from the coast) (Fig. 1), corresponding to the main distribution range of established *I. ricinus* populations in Norway (Soleng et al., 2018). Sampling sites ranged from Østfold County in southeastern Norway to Nordland County in northwestern Norway. The ticks were collected in microtubes (nymphs in pools of ten, and adults individually) and thereafter stored on crushed ice during transportation to the laboratory, where they were stored dry at -80 °C until further analyses.

### 2.2 *Detection of* Rickettsia *spp*. *and* Bartonella *spp.*

Total nucleic acids (TNA) from adult ticks (analysed individually) and pooled nymphs (analysed in pools of ten) were extracted as previously described (Paulsen et al., 2015). Samples were screened for *Rickettsia* spp. by real-time PCR targeting the citrate synthase gene (*gltA*) (Stenos et al., 2005). The *gltA* gene, a conserved housekeeping gene, was selected due to its combination of intra-species conservation and inter-species variability, enabling both broad detection of *Rickettsia* spp. and subsequent species-specific design (Ye et al., 2012). Samples testing positive in the screening were further analysed using a *R. helvetica*-specific real-time PCR targeting variable regions within *gltA.* The real-time PCR conditions for the screening were as follows: 10 μl Environmental master mix (Thermo Fisher Scientific, Baltics UAB), 0.2 μM of each primer (*gltA*-F: 5′TCGCAAATGTTCACGGTACTTT and *gltA*-R: 5′TCGTGCATTTCTTTCCATTGTG), 0.2 μM probe (5′6FAM-TGCAATAGCAAGAACCGTAGGCTGGATG-BHQ1), and RNase-free water (Thermo Fisher Scientific) was added up to 20 μl. The PCR reaction was run in a total volume of 25 μl, including 5 μl template, on a Step One Plus Real-Time PCR system (Applied Biosystems, by Thermo Fisher Scientific) with the following cycling conditions: 50°C 3 min, 95°C 5 min, 60 cycles 95°C 20 s, 60°C 40 s.

Samples that tested positive by screening were further examined with a species-specific real-time PCR to detect *R. helvetica.* This reaction included 10 μl TaqMan Environmental master mix 2.0 (Thermo Fisher Scientific, Baltics UAB), 0.2 μM of each primer (*gltA*-F 5′CCGTTTAGGTTAATAGGCTTCGG and *gltA*-R 5′CCGAGTTCCTTTAATACTTCCTTACA), 0.2 μM probe (5′6FAM-CGATCCACGTGCCGCAGTACT-BHQ1), and RNase-free water was added up to 20 μl. The PCR reaction was run in a total volume of 25, including 5 μl template, on a Step One Plus Real-Time PCR system (Applied Biosystems, by Thermo Fisher Scientific) with the following cycling conditions: 40°C 2 min, 95°C 10 min, 47 cycles 95°C 15 s, 56°C 30 s, 72°C 20 s.

*Bartonella* spp. was detected by a genus-specific real-time PCR targeting the RNA polymerase beta-subunit gene (*rpoB*) as described by Sacristan et al. (2021), with the modification that SYBRgreen PCR master mix (Thermo Fisher Scientific, Baltics UAB) was used. The reaction included 12,5 μl SYBRgreen PCR master mix (Thermo Fisher Scientific, Baltics UAB), 0.2 μM of each primer (*rpoB*-F 5′TTGAAAGTCCATATCGCAAAATT and *rpoB*-R 5′ACCTGCGTGACGGCAAAC), and RNase-free water added up to 23 μl. The PCR reaction was run in a total volume of 25 μl, including 2 μl template, on a Step One Plus Real-Time PCR system (Applied Biosystems, by Thermo Fisher Scientific), with the following cycling conditions: 95°C for 15 min, followed by 40 amplification cycles of 95°C for 30 s, 55°C for 30 s, and 72°C for 30 s. Positive control (diluted to 5 copies per microliter) (Amplirun *Bartonella henselae* DNA control; Vircell, Granada, Spain) and negative (water) control were included in each reaction, respectively. Samples yielding positive amplification in real-time PCR were subsequently analysed by conventional PCR targeting the riboflavin synthase (*ribC)* gene to confirm genus identity as described by Duodu et al. (2013). The PCR conditions were as follows: 0.2 μM of each primer (*ribC-*F 5′TAACCGATATTGGTTGTGTTGAAG and *ribC*-R 5′TAAAGCTAGAAAGTCTGGCAACATAACG), 2.5 μl dNTP-mix (10x), 2.5 μl PCR Gold Buffer, 2.5 μl MgCl_2_, 0.5 μl AmpliTaq GoldTM DNA (all reagents by Applied Biosystems, by Thermo Fisher Scientific), RNase-free water added up to 20 μl. The PCR reaction was run in a total volume of 25 μl, including 5 μl template, on a Veriti 96-well Thermal Cycler (Applied Biosystems, by Thermo Fisher Scientific). The cycling conditions were 95°C 15 min, 40 cycles 95°C 30 s, 55°C 15 s, 72°C 30s, and the final extension step 72°C 3 min. Amplicons were separated using 1.6 % agarose gel electrophoresis and visualized under UV light.

### 2.3 Statistics

Statistical analyses were performed using the Pearson Chi-square test and Clopper-Pearson exact method in STATA/SE 17.0 (STATA Corp LLC., College Station, TX, USA, 2021). P-values < 0.05 were considered statistically significant, and 95 % confidence intervals for proportions were calculated.

## 3 Results

A total of 564 adult *I. ricinus* and 3930 nymphs (tested in 393 pools) were analysed for the presence of *Rickettsia* spp. and *Bartonella* spp. (Table 1). Both pathogens were detected only in adult ticks.

**Table 1:** *Rickettsia* spp. and *Bartonella* spp. in *I. ricinus* ticks collected from counties across Norway.

County	Municipality (year)	Adults		Nymphs	
		Rickettsia spp. % (n/N)	*Bartonella* spp. % (n/N)	Rickettsia spp. % (n/N)^a^	*Bartonella* spp. % (n/N)^a^
Østfold	Hvaler (2015)	11.2 (17/152)	0.7 (1/152)	-	-
Telemark	Porsgrunn (2015)	(0/69)	1.5 (1/69)	-	-
Agder	Farsund (2015)	10.9 (5/46)	(0/46)	-	-
	Lindesnes (2015)	-	-	(0/200)	(0/200)
Rogaland	Stavanger^b^ (2016)	(0/41)	(0/41)	(0/4)	(0/4)
Vestland	Etne (2014)	-	-	(0/21)	(0/21)
	Etne (2015)	-	-	(0/101)	(0/101)
	Kinn (2016)	(0/99)	(0/99)	-	-
	Stad^c^ (2016)	(0/43)	(0/43)	-	-
Nord-Trøndelag	Nærøysund (2015)	-	-	(0/67)	(0/67)
Nordland	Brønnøy (2015)	(0/114)	0.9 (1/114)	-	-

Total		3.9 (22/564)	0.5 (3/564)	0 (0/393)^a^	0 (0/393)^a^

a N: pools of ten nymphs.

b Previously Finnøy municipality.

c Previously Eid municipality.

For *Rickettsia* spp. the overall prevalence in adult ticks was 3.9 % (22/564; 95 % CI 2.5-5.9), with positive ticks found at two locations: Hvaler in Østfold County and Farsund in Agder County, with prevalence of 11.2 % (17/152; 95 % CI 6.7-17.3) and 10.9 % (5/46; 95 % CI 3.6-23.6), respectively. Furthermore, species-specific PCR identified *R. helvetica* in 77.3 % (17/22) of the *Rickettsia*-positive adult ticks. The remaining samples could not be classified to the species level. In Østfold and Agder, 82 % (14/17) and 60 % (3/5), respectively, were confirmed as *R. helvetica*.

*Bartonella* spp. were detected exclusively in adult ticks, with an overall prevalence of 0.5 % (3/564; 95 % CI 0.1-1.66). All positive samples in the real-time PCR were confirmed by conventional PCR. Infected ticks were identified at three locations: Hvaler in Østfold County, Porsgrunn in Telemark County, and Brønnøy in Nordland County, with prevalences of 0.7 % (1/152; 95 % CI 0.02-3.61), 1.5 % (1/69; 95 % CI 0.04-7.81), and 0.9 % (1/114; 95 % CI 0.02-4.8), respectively. Co-infections with both pathogens were not found.

## 4 Discussion

This study provides the first systematic investigation of *Rickettsia* spp. and *Bartonella* spp. in questing *I. ricinus* ticks in Norway, and the first report of *Bartonella* spp. in questing ticks. *Bartonella* spp. infected ticks were found at low prevalence (≤1.5 %) in three counties, whereas a higher prevalence (11.2 and 10.9 %) of *Rickettsia* spp.-infected ticks were found in two counties. These findings demonstrate that both pathogens are present in host-seeking *I. ricinus* populations in Norway and should therefore be included in future risk assessments.

In this study, *Rickettsia* spp. were detected in *I. ricinus* ticks collected in Østfold County in eastern Norway and Agder County in southern Norway. The local prevalences of 11.2 and 10.9 % is higher than previously reported in southern Norway, where the bacterium was first detected in 2015 in ≤ 1 % of *I. ricinus* ticks collected from three sites in Agder County (Quarsten et al., 2015). Later, a more extensive study investigating *I. ricinus* nymphal ticks collected from 11 sites in southern and southeastern Norway found that although spatial variation was observed, prevalence was ≤ 5 % across sites, supporting the notion of low endemic levels of *Rickettsia* spp. in Norway (Kjaer et al., 2020). An investigation was also conducted in northern Norway, where 1 % of the ticks tested carried *R. helvetica* (Hvidsten et al., 2020). Taken together, previous Norwegian studies indicate a generally low prevalence of *Rickettsia* spp., making the observed prevalence of 11.2 and 10.9 % in Østfold and Agder notable. As tick collections in these studies were conducted in different years, the higher prevalence observed in two counties in the present research may reflect annual variation or the presence of local pathogen hotspots. Furthermore, the current study detected infection only in adult ticks. Because adult ticks have taken multiple blood meals, they are more likely to have been exposed to pathogens compared to nymphs, which may explain the high prevalence at some sites, as well as the detection only in this life stage. However, previous studies have reported infection in all tick life stages, indicating efficient transovarial transmission (Parola et al., 2005; Sprong et al., 2009). The absence of nymphal infection in the present study may therefore be coincidental, although methodological factors may have contributed to the lack of detection. Lager et al. (2017) reported higher analytical sensitivity in DNA-based assays compared with protocols using cDNA. As TNA was used in the present study, this may have influenced detection sensitivity and contributed to the lack of detection in nymphs.

Prevalence estimates of *Rickettsia* spp. in Scandinavia vary considerably across studies, ranging from 0 to 22 % (Klitgaard et al., 2019; Nielsen et al., 2004; Severinsson et al., 2010; Wallmenius et al., 2012). Several possible explanations for the varying prevalences are proposed, including the type of biotope, density of reservoir animal hosts, and tick abundance, as efficient transovarial transmission has been demonstrated (Parola et al., 2005; Sprong et al., 2009). Interestingly, a previous study suggested that the presence of *R. helvetica* in Norway may be of a more recent origin, as the prevalence observed in Norway was significantly lower than in the neighbouring countries Sweden and Denmark (Kjaer et al., 2020). Further studies, including longitudinal surveillance studies, are needed to determine why the prevalence appears to differ so markedly between studies.

In line with previous Scandinavian studies, *R. helvetica* was the predominant species in the present study, accounting for 77.3 % of the *Rickettsia*-positive ticks. Similar findings have been reported across Scandinavia, including Norway (Hvidsten et al., 2020; Kjaer et al., 2020; Klitgaard et al., 2019; Nielsen et al., 2004; Quarsten et al., 2015; Severinsson et al., 2010; Wallmenius et al., 2012), thereby supporting the results in the present study. However, a subset of the *Rickettsia*-positive samples in these studies, including our material, did not yield positive results in the *R. helvetica*-specific analysis. This may indicate the presence of other spotted fever group *Rickettsia* species, or genetic variation within *R. helvetica,* affecting assay sensitivity, as well as methodological limitations. Outside Scandinavia, *Rickettsia* spp. are widely distributed across Europe, with reported prevalence from 0.1 % to 38.5 % (Dietrich et al., 2010; Michelet et al., 2014). Although *R. helvetica* predominates in northern and central Europe, additional species such as *R. monacensis*, *R. raultii,* and *R. sloavaca* have also been identified in ticks (Arz et al., 2023; Heglasova et al., 2025). The distribution of these species is influenced by geographic, ecological, and climatic factors, as well as host availability and tick species composition. The geographic pattern observed in the present study, with detection limited to two counties and a prevalence of approximately 11 % in both locations, suggests the presence of localized foci of *Rickettsia* spp. Such spatial heterogeneity has also been reported in other European studies and may reflect local ecological conditions that favour pathogen maintenance. In this context, comparisons based on overall prevalence alone may mask important regional differences.

In the present study, *Bartonella* spp. were detected in ≤ 1.5 % of questing adult *I. ricinus* ticks. The pathogen was not detected in nymphs. Infected ticks originated from three counties (Østfold, Telemark, and Nordland), indicating a low and geographically scattered occurrence in the sampled tick population. Several studies from northern Europe have failed to detect *Bartonella* spp. in ticks; for example, a survey including 29,440 *I. ricinus* nymphs from multiple locations in Scandinavia did not detect *Bartonella* spp. (Kjaer et al., 2020). Furthermore, a study analysing 1663 ticks collected from humans in Sweden and the Aland Islands in Finland found no evidence of *Bartonella* spp. infections (Cronhjort et al., 2019). Together, these findings support the hypothesis that tick-borne transmission of *Bartonella* spp. may be uncommon in Nordic ecosystems, although the demonstration of infection in the present study indicates that transmission through tick bites cannot be excluded.

This discrepancy between the high prevalence observed in reservoir hosts in Norway (Razanske et al., 2018; Sacristan et al., 2021) and the low detection rate in questing ticks suggests that *I. ricinus* may play a limited role in maintaining *Bartonella* spp. here. However, this observation should be interpreted with caution, as transmission dynamics may influence where and how frequently the pathogen can be detected in ticks, complicating direct comparisons between host infection prevalence and prevalence in ticks. The exclusive detection in adult ticks may reflect acquisition during previous blood meals on infected hosts rather than efficient maintenance or transmission within tick populations (Krol et al., 2021). Together, these findings align with ongoing discussions regarding vector competence of ticks for *Bartonella* spp. (Boulanger et al., 2024).

Ecologically, the deer ked, an ectoparasite of cervids and a known carrier of *B*. *schoenbuchensis* (Szewczyk et al., 2017), may play a more prominent role in *Bartonella* transmission in Scandinavia. The presence of deer keds was first confirmed in Østfold County in eastern Norway in 1983 and has since expanded its distribution both southwest and north (Madslien et al., 2012; Valimaki et al., 2010). Observational data from the national biodiversity database Norway's Species Map Service indicate that deer ked populations occur in two of the counties (Østfold and Telemark) where *Bartonella*-positive ticks were detected in the present study (Norwegian Biodiversity Information Center, 2026). This spatial overlap suggests that shared cervid hosts (moose and deer) may facilitate *Bartonella* spp. exposure across arthropod species. One infected tick was also found in Nordland County, where deer keds do not occur. However, birds contribute to the long-distance dispersal of ticks, facilitating the spread of tick-borne pathogens to more northern regions. This is supported by studies of migratory birds, which have been shown to carry infected ticks over long distances (Kjelland et al., 2010; Pedersen et al., 2020). Despite the low prevalence in ticks in Norway, *Bartonella* infections in humans do occur, most notably caused by *B. henselae* (Thortveit et al., 2020). Given *B. henselae*'s strong association with cats and their fleas, these cases may reflect the established cat-flea-human route rather than tick-borne transmission. Continued surveillance, particularly studies that integrate host/ectoparasites data (including deer ked distributions), will be important for risk assessment and understanding transmission dynamics.

## 5 Conclusion

This study demonstrates that both *Rickettsia* spp. and *Bartonella* spp. occur in questing *I. ricinus* in Norway. Overall, the findings underline pronounced geographical variation in pathogen occurrence and highlight the importance of continued surveillance to better assess public health risk, pathogen distribution, and vector-host dynamics in northern ecosystems. Furthermore, future research should integrate surveillance with ecological and environmental data to improve our understanding of the factors driving pathogen emergence, maintenance and transmission, especially at the northern range limit of *I. ricinus*, where the ongoing climatic changes may influence these complex interactions.

## Data availability

Data will be available from first author or via corresponding author upon reasonable request.

## Declaration of generative AI and AI-assisted technologies in the manuscript preparation process

During the preparation of this work, the authors used Microsoft Copilot for copy editing parts of the manuscript. The authors reviewed and edited the output as needed and take full responsibility for the content of the published article.

## Funding

The study was partly funded by the 10.13039/100013276Interreg V Öresund-Kattegat-Skagerrak Programme (European Regional Development Fund) (the ScandTick Innovation project, grant number 20200422) and The Norwegian 10.13039/501100003506Ministry of Health and Care Services, the Barentsregion program (grant numbers B1412 and B1710). Funders had no role in the study design, data collection, analysis, decision to publish, or preparation of the manuscript.

## CRediT authorship contribution statement

**Hanne Kloster:** Formal analysis, Investigation, Writing – original draft, Writing – review & editing. **Camilla Stormo:** Formal analysis, Supervision, Writing – original draft, Writing – review & editing. **Andrew Jenkins:** Investigation, Resources, Supervision, Writing – review & editing. **Arnulf Soleng:** Investigation, Resources, Writing – review & editing. **Åshild Kristine Andreassen:** Conceptualization, Funding acquisition, Investigation, Resources, Writing – review & editing. **Vivian Kjelland:** Conceptualization, Funding acquisition, Project administration, Resources, Supervision, Writing – original draft, Writing – review & editing.

## Declaration of competing interest

The authors declare that they have no competing interests.
